# Some Large Lenses

**Published:** 1883-01

**Authors:** 


### SOME LARGE LENSES.

The thirty-inch objective for the great telescope of the Russian Observatory at Pulkova was lately tested at the establishment of the grinders, the Clarks, of Cambridgeport, Mass., and found to be fairly perfect. The flaw discovered before the grinding, due to imperfect cooling, has no effect on the definition, but lessens slightly the amount of light transmitted. The flaw is too slight to injure materially the efficiency of the lens, yet another block of glass, of the same size, has been ordered to be placed at the disposal of Professor Struve. For testing, the lens is mounted in a temporary telescope, forty-five feet long, and weighing, with its fittings, about seven tons. The lens weighs 450 pounds, will cost when finished $60,000, and will be for a little while the largest in the world.

The largest object-glass in use is the 26-inch lens at Washington, with a focal length of 33 feet. Its light-gathering power is 16,000 times that of the unaided eye.

The Pulkova glass will soon be excelled by that of the Lick telescope, the disk of glass for which is now in the establishment of the Clarks. It is 38 inches in diameter and 2 inches thick. When ground and polished it will be reduced to 36 inches. The glass is optically perfect. It was cast at Paris, France, where the Pulkova glass was, and weighs a little over 374 pounds. The casting occupied four days and the cooling thirty days.—*Scientific American.*

